# Integration of novel biomarkers in prospective trials: biological insights from CHOICE-01

**DOI:** 10.1038/s41392-024-02078-7

**Published:** 2024-12-05

**Authors:** Edward Christopher Dee, Puneeth Iyengar

**Affiliations:** Department of Radiation Oncology, Memorial Sloan Kettering Cancer Center, New York, NY USA

**Keywords:** Lung cancer, Cancer therapy

The recent publication of the CHOICE-01 study, reporting overall survival (OS) and published in *Signal Transduction and Targeted Therapy*,^1^ recapitulated findings from an earlier publication of the trial.^2^ The CHOICE-01 investigators prospectively randomized patients with locally advanced (IIIB or IIIC) or metastatic non-small-cell lung cancer (NSCLC) without targetable *EGFR* or *ALK* mutations to chemotherapy with or without toripalimab, a humanized PD-1 directed IgG4 antibody, and demonstrated an OS improvement associated with toripalimab.

**Fig. 1:**
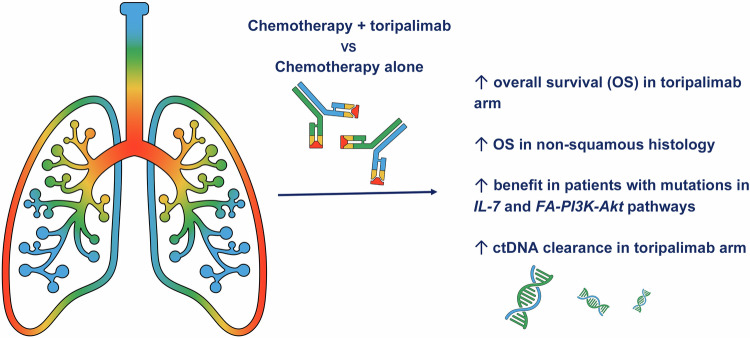
Graphic summary of the CHOICE-01 trial. This figure summarizes the results of the CHOICE-01 trial, which randomized patients with advanced (IIIB or IIIC) or metastatic non-small-cell lung cancer without targetable EGFR or ALK mutations to chemotherapy with or without toripalimab

In this most recent publication of CHOICE-01, at a median follow-up of 21.2 months, toripalimab was associated with improved OS compared with placebo (median OS 23.8 vs 17.0 months, HR 0.69, 95%CI 0.57–0.93) (Fig. 1).^1^ These findings are a welcome addition to the therapeutic landscape of lung cancer – particularly for those without currently actionable mutations – given lung cancer’s role as a leading cause of cancer death globally.

One limitation of the study may be the control arm. Chemotherapy alone is standard-of-care for only a minority of patients with advanced NSCLC for whom immunotherapy is intolerable, with prior immunotherapy trials having set the standard.^3,4^ Comparing toripalimab to another PD-1 or PD-L1 inhibitor may have yielded findings that build more clearly upon standard therapy. Furthermore, the study was completed in multiple institutions in China. Further validation in more globally diverse geographic settings may improve the generalizability of the trial’s findings.

The CHOICE-01 study is nonetheless groundbreaking for multiple reasons, beyond the demonstration of an OS benefit. The first is the demonstration of benefit only in the non-squamous NSCLC cohort (median OS 27.8 vs. 15.9 months, HR 0.49, 95%CI 0.35–0.69), and the lack of benefit among patients with squamous cell carcinoma (SCC, median OS 19.6 vs. 18.1 months, HR 1.09, 95%CI 0.77–1.56). The KEYNOTE-407 trial showed benefit associated with pembrolizumab in metastatic lung SCC (HR 0.64; 95%CI, 0.49–0.85),^3^ although this benefit may be smaller than among patients with non-squamous NSCLC, as in KEYNOTE-189 (HR 0.49; 95% CI, 0.38–0.64),^4^ on the caveat of the limitations of cross-trial comparisons.

Additionally, the authors find that tumor mutational burden (TMB) was associated with improved PFS in the toripalimab group, but only among patients with non-squamous SCC. The findings of CHOICE-01 highlight that SCC is distinct from adenocarcinoma, underscoring the need for trials and guidelines that are informed by such histological distinctions. Future work – not only in systemic therapy but in radiation and multimodal studies – should similarly explore SCC and non-squamous NSCLC as separate diseases.

Second, the CHOICE-01 trial is among the first to rigorously incorporate modern biomarkers in a prospective therapeutic trial, in addition to classical biomarkers such as tumor mutational burden (TMB) and PD-L1 expression. Whole exome sequencing conducted on a majority of tissue samples demonstrated patients with mutations in the *IL-7* and *FA-PI3K-Akt* pathways benefited to a greater extent from toripalimab treatment. In parallel, at C3D1, the authors found a greater proportion of ctDNA-negative patients in the toripalimab arm compared with the placebo arm, and that 82.1% of patients with no detectable ctDNA in the toripalimab arm vs 58% in the placebo arm showed RECIST-defined response. These prospective findings add to a growing body of work demonstrating the utility of ctDNA in prognosticating outcomes in NSCLC.^5^

Future work must explore how to integrate ctDNA-based findings in guiding not just treatment response but treatment selection, escalation, and de-escalation. Although *FA-PI3K-Akt* and *IL-7* alterations detected by ctDNA may prognosticate improved response, would such findings favor the use of toripalimab, and perhaps more critically, would the converse hold true? Future research must also explore how biomarkers such as ctDNA can guide local therapies such as radiation, independently and in conjunction with systemic therapies. The authors hypothesize that novel biomarkers may guide systemic treatment selection; can the same be said of patient selection for local ablative therapy in the setting of metastatic disease?

The findings of CHOICE-01 also open further avenues of inquiry in the treatment of metastatic disease. The greatest benefit appears to be among patients with IVA (HR 0.72, 95%CI 0.511–1.016) and IVB disease (HR 0.67, 95%CI 0.458–0.996), compared with IIIB/IIIC (HR 0.79, 95%CI 0.398–1.653). The benefit may be greater amongst patients with bone metastases (HR 0.74, 95%CI 0.490–1.132) compared with patients with liver metastases (HR 0.97, 95%CI 0.499–1.986). These latter findings are perhaps due more to poor outcomes associated with liver metastases, but future work may explore biological underpinnings of metastatic tropism and the association with treatment response. Does local therapy have a role to play in potentiating response to immunotherapy among patients with liver metastases? Furthermore, the role of local therapy for metastatic disease in the context of combination chemotherapy-immunotherapy merits further prospective evaluation. In the CHOICE-01 toripalimab arm, would radiotherapy to synchronous oligometastatic or oligopersistent lesions have improved PFS to an even greater degree?

Relatedly, the biomarker findings of CHOICE-01 yield potential implications for the timing of local therapy. Although the cohort that received immunotherapy in the phase II/III trial LU-002 showed no benefit with metastasis-directed therapy among patients with oligometastatic NSCLC, future work should explore how to improve biologically-guided patient selection in the context of systemic advances. As we gain insight into the role of ctDNA in prognosticating response, we must also explore whether the timing of ctDNA clearance may prove instructive for the timing of local therapy. Does ctDNA have a role to play not just in patient selection but in the incorporation and timing of consolidative radiation for patients with metastatic NSCLC?

The CHOICE-01 trial is groundbreaking in the thorough integration of novel biomarkers in a modern systemic therapy trial for SCC and non-squamous NSCLC. In addition to demonstrating an OS benefit for toripalimab, the study is an exemplar of how modern trials can ask multiple biologically driven questions. CHOICE-01 is yet another piece of evidence supporting treating SCC as a separate entity, for example. Perhaps more importantly, the demonstration of the predictive power of ctDNA – both ctDNA clearance and ctDNA-determined mutations – may advance future questions about how biology can guide treatment. The next steps should build on this study to evaluate whether novel biomarkers may not just prognosticate but guide patient selection, treatment choice, and timing of local therapy. CHOICE-01 answers some questions and asks many more, potentially facilitating further personalization of cancer treatment.
